# Estimated glucose disposal rate and risk of first myocardial infarction in people with diabetes

**DOI:** 10.1186/s40842-026-00294-4

**Published:** 2026-05-25

**Authors:** Linn Glynn, Martina Persson, Tomas Andersson, Robin Hofmann, Thomas Nyström

**Affiliations:** 1 Department of Clinical Science and Education, Södersjukhuset, Karolinska Institutet, Sjukhusbacken 10, 118 83, Stockholm, Sweden; 2 Institute of Environmental Medicine, Karolinska Institutet, Stockholm, Sweden

**Keywords:** Diabetes mellitus, Insulin resistance, Cardiovascular complications, Myocardial infarction, Estimated glucose disposal rate

## Abstract

**Background:**

The estimated glucose disposal rate (eGDR), calculated from glycated hemoglobin (HbA1c), hypertension status and BMI, is a proxy for insulin resistance. We examined the association between eGDR and the risk of first myocardial infarction (MI) and post-MI mortality in individuals with type 1 and type 2 diabetes.

**Methods:**

Using nationwide health registry data (2006–2020), we identified individuals with type 1 and type 2 diabetes. Follow-up started at the first time a complete eGDR could be calculated. eGDR was then handled as a time-updated exposure until first MI, with person-time assigned according to the most recent available value. Among those with MI, follow-up for post-MI mortality started at the event, and covariates were fixed at their last value before MI. Hazard ratios (HRs) with 95% CIs were estimated using Cox regression with adjustment for blood lipids, albuminuria, estimated glomerular filtration rate and physical activity.

**Results:**

We included 46,155 individuals with type 1 diabetes and 570,230 individuals with type 2 diabetes (both 43% women). During a median follow up of 7.7 person-years, 1997 (4.3%) and 34,237 (6.0%) experienced first MI, respectively. Adjusted HRs (95% CIs) for first MI across eGDR categories ≤4, 4–6, 6–8, and ≥8 mg/kg/min) were: 3.73 (3.35–4.16), 3.09 (2.85–3.35), 2.40 (2.15–2.69) and 0.96 (0.79–1.17) for type 1 diabetes, respectively. Corresponding HRs for type 2 diabetes were 1.71 (1.62–1.82), 1.54 (1.46–1.62), 1.43 (1.35–1.51) and 1 (reference). Similar associations were observed for post-MI mortality, however non-significant across all eGDR categories.

**Conclusions:**

Lower eGDR, reflecting greater insulin resistance, was associated with a higher risk of first MI, with a stronger association observed in type 1 diabetes. These findings support insulin resistance as a potential modifiable cardiovascular risk factor in people with diabetes.

**Graphical Abstract:**

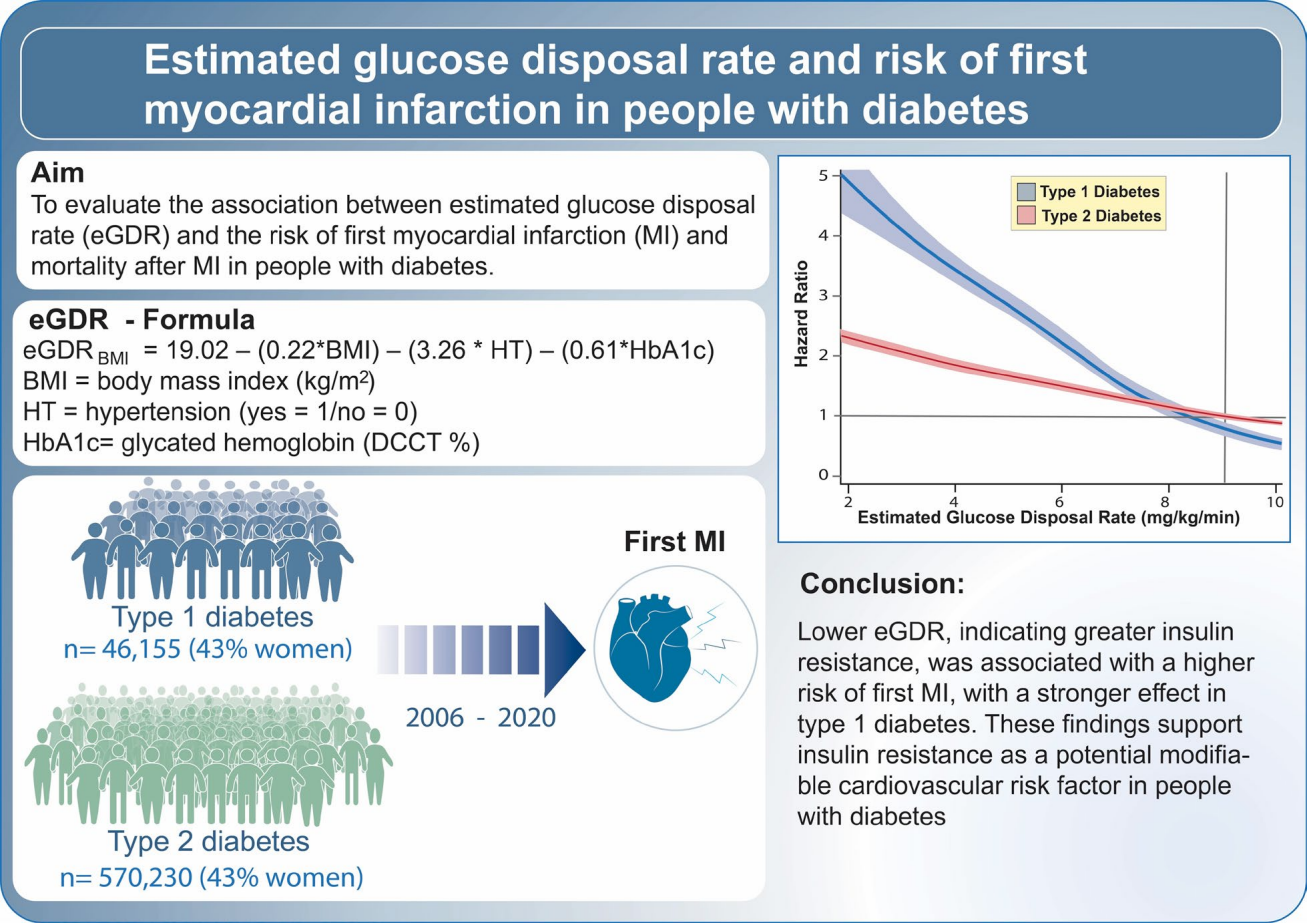

**Supplementary Information:**

The online version contains supplementary material available at 10.1186/s40842-026-00294-4.

## Research insights

What is currently known about this topic?

What is the key research question?

What is new?

How might this study influence clinical practice?

Myocardial infarction is a major complication of diabetes. Estimated glucose disposal rate is a proxy of insulin resistance. Its contribution to cardiovascular risk across diabetes types remains unclear.

Does estimated glucose disposal rate predict first myocardial infarction in people with diabetes?

Lower estimated glucose disposal rate predicts first myocardial infarction. The association was stronger in type 1 than in type 2 diabetes.

Estimated glucose disposal rate may identify individuals at high risk of myocardial infarction.

**Supplementary Information:**

The online version contains supplementary material available at 10.1186/s40842-026-00294-4.

## Background

Cardiovascular disease (CVD) is the leading cause of premature death in individuals with diabetes [1]. The coexistence of type 1 diabetes and the metabolic syndrome, known as “double diabetes”, poses a major clinical challenge [2]. This phenotype is characterized by features typical of type 2 diabetes: hypertension, dyslipidemia, abdominal obesity, and insulin resistance, and is associated with increased risk of CVD [2]. Rising obesity rates among people with type 1 diabetes are expected to further increase its prevalence [3].

Insulin resistance is central for the development of CVD in individuals with type 2 diabetes and in the general population [4], and previous results from our group indicate it may be of importance also in type 1 diabetes [5]. People with diabetes of any type have a two-fold risk of coronary artery disease, including myocardial infarction (MI), compared to the general population [6], with some evidence suggesting an even higher relative risk in type 1 diabetes [7]. Although survival after a first MI has improved over the past decade in people with type 2 diabetes, no such improvement has been observed in type 1 diabetes [8]. The reason for this disparity remains unclear.

The euglycemic hyperinsulinemic clamp is the gold standard for assessment of insulin resistance, but it is an invasive method and not suitable for large-scale epidemiological studies or routine care [9]. As an alternative, the estimated glucose disposal rate (eGDR) based on glycated hemoglobin (HbA1c), hypertension status, and waist circumference, waist to hip ratio or body mass index (BMI) has been suggested as a proxy of insulin resistance [10, 11]. eGDR was developed and validated against the clamp method in type 1 diabetes [10], and has later shown to correlate with clamp-derived measures of insulin resistance in a small cohort of individuals with type 2 diabetes [11].

Lower eGDR (i.e. higher insulin resistance) has been associated with microvascular complications [12], coronary artery disease [13] and mortality [5] in people with type 1 diabetes, and with stroke [11], all-cause and cardiovascular death [14], and mortality after coronary artery bypass grafting in type 2 diabetes [15]. However, no previous studies have investigated the association of eGDR and risk of first MI in individuals with diabetes, nor whether the association differs between individuals with type 1 diabetes and type 2 diabetes.

The aim of this study was to investigate if lower eGDR is associated with an increased risk of first MI in people with diabetes and to explore potential differences between individuals with type 1 and type 2 diabetes. Direct comparison of cardiovascular outcomes in type 1 and type 2 diabetes may clarify whether risk differs by diabetes type, with implications for cardiovascular risk assessment and preventive care. We hypothesized that lower eGDR (reflecting higher insulin resistance) would be associated with a higher risk of first MI, particularly in those with type 1 diabetes.

## Methods

### Study design and data sources

This nationwide observational cohort study used data from several Swedish national health care registers: the National Diabetes Register (NDR) [16], the National Patient Register (NPR) [17], the National Cause of Death Register [18], the Prescribed Drug Register (PDR) [19] and Longitudinal Integration Database for Health Insurance and Labour Market Studies (LISA) [20]. The Swedish personal identification number is unique to all Swedish residents and enables individual-level linkage between different national health registries.

The NDR includes the majority of individuals with known diabetes mellitus in Sweden, and diagnoses are made by trained physicians according to criteria from the American Diabetes Association [21] and registered as International Classification of Diseases (ICD) codes in the NDR. People with Latent Autoimmune Diabetes in Adults (LADA) are classified as type 1 diabetes [22]. In 2024, the population coverage of individuals with type 2 diabetes in the NDR was estimated at 83% based on a comparison with individuals aged 50–79 who had records of glucose-lowering medication in the PDR [22]. This assessment excluded individuals outside the specified age group as well as those treated with diet alone. For children and adolescents with type 1 diabetes, the estimated population coverage was 97% compared with ICD codes in the NPR [22]. Data in the NDR is collected and transferred to the register at each outpatient visit to specialist clinics or primary care.

The NPR collects diagnoses according to the ICD coding system from inpatient care with nationwide coverage since 1987 [17]. Data from visits to specialized outpatient care have been included since 2001 and comprise non-admitted medical services delivered by specialist physicians or multidisciplinary teams; primary care is not included. The positive predictive value of an MI diagnosis in the NPR was estimated to over 90% [17].

The National Cause of Death Register provides information on time of death and causes of death for all deceased individuals registered in Sweden [18]. The PDR provides information on dispensed prescriptions since 2006 [19]. LISA provides information on socioeconomic status categorized by education, income, and employment [20].

### Inclusions, exposures and outcomes

Adults (≥ 18 years) with type 1 and type 2 diabetes living in Sweden were included at first complete registration of eGDR_BMI_ (i.e., HbA1c, hypertension status, and BMI) in the NDR between 1st January 2006 and 31st December 2016. For those with ICD codes of unspecified type of diabetes, epidemiological criteria were used. Type 1 diabetes was defined as a diagnosis at age ≤ 30 years and requiring treatment with insulin and type 2 diabetes was defined as treatment with diet, with or without oral antihyperglycemic agents, or treatment with insulin with or without oral antihyperglycemic agents and diagnosis at age ≥ 40 years [16]. Those with other specified types of diabetes (i.e. Maturity Onset Diabetes in the Young, secondary diabetes or cystic fibrosis-related diabetes) or unspecified type of diabetes that could not be defined by epidemiological criteria or individuals lacking covariates in the eGDR formula were excluded. Individuals with a previous diagnosis of MI before first complete eGDR-registration were also excluded.

eGDR_BMI_ (mg/kg/min) was calculated using the following formula [11]:

$$\begin{aligned}\rm eGDR_{BMI}=&19.02 - (0.22^*BMI) \cr&-(3.26^ * HT) -(0.61^*HbA1c)\end{aligned}$$

BMI = body mass index (kg/m^2^), HT = hypertension (yes = 1/no = 0), and HbA1c = HbA1c (DCCT %).

BMI was calculated based on self-reported or measured height and weight. Hypertension was defined as blood pressure > 140/90 or current use of antihypertensive agents. Individuals were considered hypertensive from the date of their first recorded diagnosis and onward. Analyses of HbA1c levels were performed at certified local laboratories and reported according to the International Federation of Clinical Chemistry standard (mmol/mol). HbA1c values were converted to standard values according to the National Glycohemoglobin Standardization Program [23].

Individuals were categorized into four groups according to eGDR-levels consistent with previous studies [5, 12]: ≤4, 4–6, 6–8, and ≥8 mg/kg/min. Higher eGDR values reflected greater insulin sensitivity. Individuals with type 2 diabetes and eGDR *≥* 8 mg/kg/min were used as the reference group in all analyses to provide a metabolically comparable baseline to type 1 diabetes and because higher insulin sensitivity was hypothesized to be associated with lower MI risk.

Follow-up started at the first time a complete eGDR could be calculated. Thereafter, eGDR was updated over time, and follow-up time was allocated according to the most recent available eGDR category. If the components of the eGDR formula were not recorded at the same visit, the most recent previously recorded value was used. Follow-up time was allocated according to the most recent available eGDR category in a time-updated framework, ensuring that exposure status was assigned prospectively as new measurements became available.

The primary outcome was time to first MI, retrieved from the NPR (ICD-codes are specified in Supplemental Table S1). Last date of follow-up for MI was 31st December 2021. Individuals experiencing an MI were subsequently followed until death (secondary outcome), emigration or end of study (31st May 2022).

### Statistical analysis

Continuous variables were summarized as means and standard deviations (± SD) and categorical variables as numbers and percentages. Incidence rates were reported overall and stratified by sex, and subsequentially standardized to the internal age and sex distribution of the whole study population.

Crude and adjusted hazard ratios (HRs) with 95% CIs for first MI and all-cause mortality after MI were estimated using Cox regression. Cox models used calendar time as the underlying time scale. Sex was included as a stratification factor, and age was modelled as a time-updated third-degree polynomial. All components of eGDR and other covariates in the multivariate adjusted model were handled as time-updated variables, with the most recent value carried forward until new information was recorded. For the analysis of all-cause mortality post MI, the time scale was time since MI, and covariates were fixed at their last value before the event. In addition, eGDR was modelled as a continuous variable using restricted cubic splines to explore potential non-linear relationships.

Potential confounders were considered based on clinical knowledge and prior literature [24]. Candidate confounders were evaluated using a change-in-estimate approach, comparing unconstrained and constrained models for the eGDR coefficients via likelihood ratio testing [25]. In these univariable assessments, each covariate was evaluated using complete cases only. Covariates that materially altered the eGDR estimates (*p* < 0.10) were included in the final multivariable model, where missing values were accommodated using an indicator approach to retain individuals and person-time. Results from the covariate-by-covariate change-in-estimate assessments, including analyses of age at diabetes onset and diabetes duration, are presented in the Supplemental material. In the final model, sex was used as a stratification factor and age was modelled as a time-updated third-degree polynomial; the model was further adjusted for triglycerides, low-density lipoprotein (LDL) cholesterol, high-density lipoprotein (HDL) cholesterol, albuminuria, estimated glomerular filtration rate (eGFR), and self-reported physical activity [24]. All CIs were calculated using Wald-type methods. All analyses were performed using SAS software, version 9.4 (TS1M8) (SAS Institute Inc., Cary, NC, USA).

## Results

### Study population

A total of 616,385 individuals matching the inclusion and exclusion criteria were identified from 1st January 2006 to 31st December 2020 (Supplementary Figure S1). Of these, 46,155 (7.5%) individuals had type 1 diabetes, and 570,230 (92.5%) individuals had type 2 diabetes, 43% women in both groups (Table 1). Baseline characteristics stratified by diabetes type are presented in Table 1. Individuals with type 1 diabetes had a mean age of 37 years, mean HbA1c 64.3 mmol/mol (8.0%) and diabetes duration of 18.4 years (Table 1). Individuals with type 2 diabetes had a mean age of 65 years, mean HbA1c 54.3 (7.1%) mmol/mol and mean diabetes duration 4.4 years (Table 1). A greater proportion of individuals with type 2 diabetes had prior cardiac interventions (angiography, percutaneous cardiac intervention and coronary artery bypass grafting), more comorbidities and were treated with more cardiovascular preventative medication those with type 1 diabetes (Table 1).

**Table 1:** Baseline characteristics by diabetes type

Baseline characteristics	Type 1 diabetes	Type 2 diabetes
n	46 155	570 230
Age, years, mean (± SD)	37.3 (15.7)	64.6 (12.7)
Women, n (%)	20 031 (43.4)	247 363 (43.4)
Active smoker, n (%)	6579 (14.3)	85 391 (15.3)
BMI, kg/m^2^, mean (± SD)	25.0 (4.0)	30.4 (5.7)
**Marital status**,** n (%)**		
Married	13 893 (30.1)	302 347 (53.0)
Separated/Single	31 643 (68.6)	202 006 (35.4)
Widowed	619 (1.3)	65 869 (11.6)
**Education**,** n (%)**		
Elementary school	13 880 (30.4)	207 843 (37.2)
Upper secondary school	19 943 (43.7)	243 758 (43.6)
College level	11 834 (25.9)	107 472 (19.2)
**Blood lipids**,** mean (± SD)**		
Total cholesterol, mmol/L	4.70 (0.97)	5.03 (1.15)
HDL-cholesterol, mmol/L	1.57 (0.47)	1.26 (0.39)
LDL-cholesterol, mmol/L	2.65 (0.82)	2.95 (1.01)
Triglycerides, mmol/L	1.14 (0.87)	1.98 (1.48)
Systolic blood pressure, mmHg, mean ± SD	125 (16)	138 (17)
Diastolic blood pressure, mmHg, mean ± SD	73 (9)	79 (10)
Creatinine, µmol/L, mean ± SD	78.4 (42.4)	78.4 (28.4)
eGFR, ml/min, mean ± SD	96.9 (28.5)	83.6 (24.5)
**Prior interventions**,** n (%)**		
Coronary angiography	67 (0.1)	11 353 (2.0)
Revascularization	405 (0.9)	15 596 (2.7)
PCI	172 (0.4)	8305 (1.5)
CABG	254 (0.6)	8004 (1.4)
**History of comorbidities**,** n (%)**		
Stroke	669 (1.4)	31 128 (5.5)
Heart failure	390 (0.8)	24 934 (4.4)
Atrial fibrillation	342 (0.7)	39 849 (7.0)
Peripheral artery disease	126 (0.3)	2864 (0.5)
Hypertension	5194 (11.3)	169 688 (29.8)
COPD	178 (0.4)	16 850 (3.0)
Cancer	505 (1.1)	35 136 (6.2)
Cancer (within 2 years)	189 (0.4)	10 410 (1.8)
**Treatments previous 180 days**,** n (%)**		
Statin	7851 (17.0)	229 587 (40.3)
ASA	4821 (10.4)	142 818 (25.0)
P2Y12	243 (0.5)	10 892 (1.9)
Betablocker	3834 (8.3)	194 297 (34.1)
ACEi/ARB	9396 (20.4)	278 229 (48.8)
Diabetes onset age, years, mean (± SD)	18.5 (10.8)	60.1 (12.8)
Diabetes duration, years, mean (± SD)	18.4 (14.8)	4.4 (6.4)
HbA1c (mmol/mol), mean (± SD)	64.3 (16.4)	54.3 (16.3)
HbA1c (DCCT %), mean (± SD)	8.0 (3.7)	7.1 (3.6)
**Albuminuria**,** n (%)**		
None	38 287 (84.4)	414 970 (79.5)
Microalbuminuria	4573 (10.1)	75 399 (14.4)
Macroalbuminuria	2362 (5.2)	29 200 (5.6)
**Physical activity**,** times per week**,** n (%)**		
Never	3977 (8.9)	83 553 (15.7)
<1 times/week	6405 (14.4)	68 790 (12.9)
1–2 times/week	11 753 (26.4)	109 278 (20.5)
3–5 times/week	12 303 (27.7)	114 982 (21.6)
Daily	10 029 (22.6)	156 118 (29.3)
**Diabetes treatment last 180 days**,** n (%)**		
Diet only	2357 (5.1)	206 096 (36.1)
Insulin all	43 603 (94.5)	92 897 (16.3)
Insulin long acting	36 247 (78.5)	62 372 (10.9)
Insulin fast acting	40 378 (87.5)	27 825 (4.9)
Metformin	1411 (3.1)	287 705 (50.5)
Sulfonylurea	164 (0.4)	61 154 (10.7)
DPP-4 inhibitor	52 (0.1)	10 268 (1.8)
GLP-1 receptor agonist	49 (0.1)	3465 (0.6)
SGLT2-inhibitor	13 (0.0)	2585 (0.5)

Baseline characteristics at first complete estimated glucose disposal rate measurement in individuals with type 1 diabetes and type 2 diabetes

ASA, Acetylsalicylic acid; ACE-I, Angiotensin-converting-enzyme inhibitor; ARB, Angiotensin II receptor-blocker; BMI, Body mass index; CABG, Coronary artery bypass graft; COPD, chronic obstructive lung disease; DPP-4 inhibitor, Dipeptidylpeptidas inhibitor; eGFR, Estimated glomerular filtration rate; GLP-1, Glucagon like peptide-1 receptor agonist; HbA1c, Glycated haemoglobin 1c; HDL-Cholesterol, High-density lipoprotein-Cholesterol; LDL-Cholesterol, Low-density lipoprotein-Cholesterol, PCI, percutaneous coronary intervention; P2Y12, P2Y12-inhibitors; SGLT2-inhibitor, Sodium-glucose transport 2-inhibitor

Baseline characteristics across eGDR categories are presented in Supplementary Table S2 and S3. Among individuals with type 1 diabetes, those with eGDR ≤4 mg/kg/min tended to have higher BMI, higher total and LDL cholesterol, triglycerides, blood pressure, and HbA1c than individuals with eGDR ≥8 mg/kg/min, and they were more frequently treated with metformin (Supplementary Table S2). Individuals with lower eGDR were slightly older, had a higher proportion of prior cardiac interventions, more comorbidities, more cardiovascular preventative medication compared to those with higher eGDR. Duration of diabetes was longer for the two lowest eGDR-categories.

For individuals with type 2 diabetes, those with eGDR ≤4 mg/kg/min generally had higher BMI, blood pressure and HbA1c and more often heart failure and treatment with insulin than those with eGDR ≥8 mg/kg/min (Supplementary Table S3). In contrast, LDL-cholesterol appeared highest for individuals with eGDR ≥8 mg/kg/min. There was no clear pattern regarding differences in prior cardiac interventions, diabetes duration or age of onset for individuals with type 2 diabetes. Proportions of cardiovascular preventative medication were similar in the three lowest eGDR-categories. The prevalence of micro- and macroalbuminuria showed an increasing pattern with decreasing eGDR in individuals with both diabetes types. For both diabetes types, college level education was slightly more common for those with eGDR ≥8 mg/kg/min.

The median time between eGDR updates was 109 days (IQR 34–223). For 58.4% of eGDR updates, all required component information was available from the same date. In the remaining updates, where at least one component was carried forward from an earlier date, the median age of the oldest component was 160 days (IQR 64–324).

### eGDR and risk of first MI

After a median follow-up of 7.7 person-years, 1,997 MI (4.3%) cases occurred in individuals with type 1 diabetes, and 34,237 (6.0%) MI cases in those with type 2 diabetes. The crude incidence rates for first MI were higher in individuals with type 2 diabetes (Table 2). After age- and sex-standardization, incidence rates were significantly higher among those with type 1 diabetes (Table 2). For individuals with type 2 diabetes, sex-stratified rates showed higher incidence rates in men than in women, but no such difference was observed in individuals with type 1 diabetes (Supplementary Table S4). For type 1 diabetes, age and sex standardized incidence rates of first MI per 1000 patient-years were 13.9, 13.0, 9.2, and 5.2 across eGDR categories ≤4, 4–6, 6–8, and ≥8 mg/kg/min, respectively (Table 2). Corresponding incidence rates for individuals with type 2 diabetes were 9.1, 7.3, 6.2, and 4.5 per 1000 person-years (Table 2).

**Table 2:** Estimated glucose disposal rate and risk of first myocardial infarction

	eGDR (mg/kg/min)	MI cases (n)	p-yrs	Crude IR per 1000 p-yrs	Age and Sex IR per 1000 p-yrs	HR crude	HR sex and age^*^	HR adj^†^
T1D	≤4	441	56 987	7.7 (7.1–8.5)	13.9 (11.8–16.4)	2.15 (1.94–2.39)	4.24 (3.81–4.72)	3.73 (3.35–4.16)
	4–6	1 049	169 454	6.2 (5.8–6.6)	13.0 (11.9–14.3)	1.70 (1.57–1.84)	2.83 (2.61–3.06)	3.09 (2.85–3.35)
	6–8	396	125 561	3.2 (2.9–3.5)	9.2 (8.0-10.6)	0.86 (0.77–0.96)	1.95 (1.74–2.18)	2.40 (2.15–2.69)
	≥8	111	204 487	0.5 (0.5–0.7)	5.2 (2.9–9.2)	0.15 (0.12–0.18)	0.70 (0.58–0.86)	0.96 (0.79–1.17)
T2D	≤4	8 013	950 652	8.4 (8.3–8.6)	9.1 (8.9–9.3)	2.43 (2.29–2.56)	2.20 (2.08–2.32)	1.71 (1.62–1.82)
	4–6	17 320	2 080 940	8.3 (8.2–8.5)	7.3 (7.2–7.5)	2.39 (2.27–2.52)	1.71 (1.62–1.80)	1.54 (1.46–1.62)
	6–8	7 450	1 006 678	7.4 (7.2–7.6)	6.2 (6.1–6.4)	2.13 (2.01–2.25)	1.43 (1.35–1.51)	1.43 (1.35–1.51)
	≥8	1 454	406 647	3.6 (3.4–3.8)	4.5 (4.2–4.7)	1 (reference)	1 (reference)	1 (reference)

Crude and age and sex standardized incidence rates (IR) of first myocardial infarction (MI) per 1000 person years. HR with 95% CIs of first MI, according to diabetes type (T1D, type 1 diabetes; T2D, type 2 diabetes) and level of estimated glucose disposal rate (eGDR) in mg/kg/min

* Adjusted for sex and age

† Adjusted for sex and age, physical activity, High-density lipoprotein-Cholesterol, Low-density lipoprotein-Cholesterol, triglycerides, albuminuria and estimated glomerular filtration rate

**Fig. 1:**
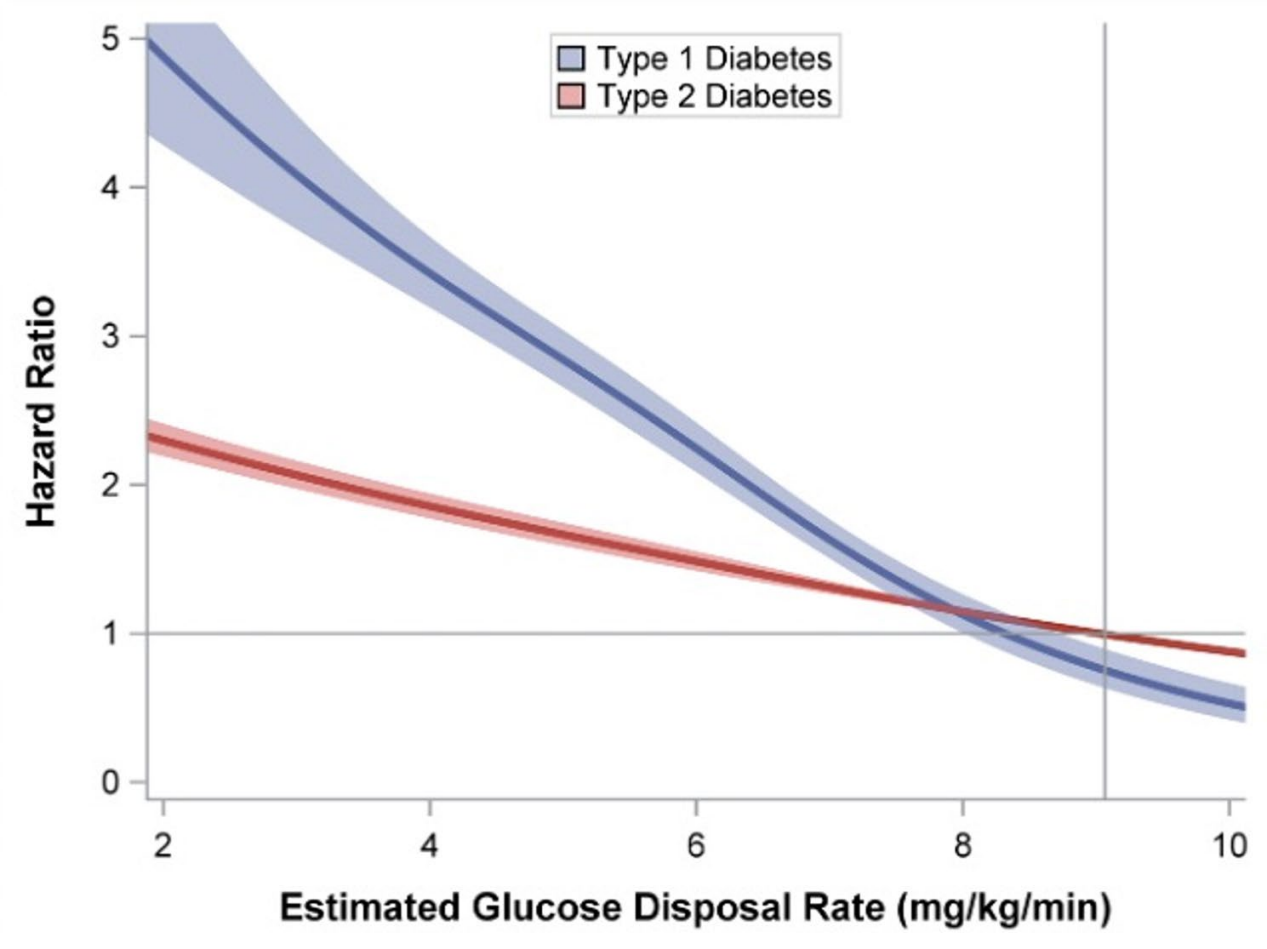
Association between estimated glucose disposal rate and first myocardial infarction in type 1 and type 2 diabetes. Restricted cubic spline with knots at estimated glucose disposal rate (eGDR) values 4, 6 and 8 mg/kg/min showing age- and sex-adjusted hazard ratios (HRs) for first myocardial infarction in individuals with type 1 diabetes and type 2 diabetes

Lower eGDR was inversely associated with a higher HR of first MI in both type 1 and type 2 diabetes (Fig. 1). Compared with individuals with type 2 diabetes in the highest eGDR category, adjusted HRs with 95% CIs for type 1 diabetes were: 3.73 (3.35–4.16), 3.09 (2.85–3.35), 2.40 (2.15–2.69), and 0.96 (0.79–1.17) in categories eGDR ≤4, 4, 4–6, 6–8 and ≥8 mg/kg/min, respectively (Table 2). Corresponding HRs for type 2 diabetes were 1.71 (1.62–1.82), 1.54 (1.46–1.62), 1.43 (1.35–1.51), and 1 (reference) (Table 2).

The relative gradient of risk across eGDR categories was steeper in type 1 diabetes than in type 2 diabetes. HRs after adjustment for sex, age and each confounder individually as well as separate adjustment for age of onset of diabetes and diabetes duration are presented in Supplementary Table S5. After adjustment for diabetes duration there was no significant difference in risk of first MI between diabetes types for those with eGDR ≤4 mg/kg/min or eGDR 4–6 mg/kg/min. Individuals with type 1 diabetes and eGDR 6–8 mg/kg/min and ≥8 mg/kg/min had a lower risk than individuals with type 2 diabetes in equivalent categories (Supplemental Table S5). A similar pattern was observed for age of onset of diabetes.

### eGDR and mortality after first MI

After a median follow-up of 3.4 person-years after the first MI, 918 deaths (45.9%) occurred among individuals with type 1 diabetes and 18,584 deaths (54.2%) among those with type 2 diabetes. Mortality rates were higher for people with type 2 diabetes compared to people with type 1 diabetes (Table 3; Supplementary Figure S2). Age- and sex-standardized mortality rates per 100 person-years for type 1 diabetes were: 9.2, 8.9, 8.8, and 3.1 deaths per 100 person-years for eGDR categories ≤4, 4–6, 6–8, and ≥8 mg/kg/min, respectively (Table 3). Corresponding rates for individuals with type 2 diabetes were 11.8, 12.3, 15.7, and 7.8 per 100 person-years across eGDR categories ≤4, 4–6, 6–8, and ≥8 mg/kg/min, respectively (Table 3). Sex-stratified analysis showed higher mortality rates for women across all eGDR-categories in individuals with type 2 diabetes, no sex-differences were observed in individuals with type 1 diabetes (Supplementary Table S6).

**Table 3:** Estimated glucose disposal rate and post-myocardial infarction mortality

	eGDR (mg/kg/min)	n dead	p-yrs	Mortality rate per 100 p-yrs	Age and sex mortality rate per 100 p-yrs	HR sex and age^*^	HR adj^†^
T1D	≤4	209	2 281	9.2 (8.0-10.5)	24.4 (17.7–33.6)	2.92 (2.49–3.42)	2.09 (1.78–2.45)
	4–6	503	5 622	8.9 (8.2–9.8)	16.7 (14.7–19.0)	1.97 (1.75–2.22)	1.57 (1.39–1.77)
	6–8	181	2 065	8.8 (7.6–10.1)	15.4 (12.8–18.4)	1.75 (1.49–2.07)	1.55 (1.31–1.83)
	≥8	25	817	3.1 (2.1–4.5)	10.4 (5.7–19.3)	1.16 (0.78–1.74)	1.11 (0.74–1.67)
T2D	≤4	4 219	35 779	11.8 (11.4–12.2)	14.1 (13.7–14.6)	1.47 (1.35–1.60)	1.18 (1.09–1.29)
	4–6	9 302	75 441	12.3 (12.1–12.6)	11.2 (11.0-11.4)	1.12 (1.03–1.21)	1.02 (0.94–1.11)
	6–8	4 435	28 280	15.7 (15.2–16.2)	12.7 (12.4–13.1)	1.21 (1.12–1.32)	1.17 (1.08–1.28)
	≥8	628	8 062	7.8 (7.2–8.4)	10.8 (9.9–11.7)	1 (reference)	1 (reference)

Crude and age- and sex-standardized mortality rates after first myocardial infarction (MI) per 100 person years. HRs with 95% CIs of all-cause death after first MI, according to diabetes type (T1D, type 1 diabetes; T2D, type 2 diabetes) and level of estimated glucose disposal rate (eGDR) in mg/kg/min

* Adjusted for sex and age

† Adjusted for sex and age, physical activity, High-density lipoprotein-Cholesterol, Low-density Lipoprotein-Cholesterol, triglycerides, albuminuria and estimated glomerular filtration rate

Among people with type 1 diabetes, there was a trend towards higher post-MI mortality with lower eGDR, although CIs overlapped between intermediate categories (Table 3). Individuals in the lowest eGDR group (≤ 4 mg/kg/min) had a significantly higher mortality risk compared with the highest eGDR category for both diabetes types (Table 3). In type 2 diabetes, multivariate-adjusted HRs for post-MI mortality were generally lower than those observed in type 1 diabetes, with the highest risks observed in those with eGDR ≤4 and 6–8 mg/kg/min (Table 3).

## Discussion

In this nationwide, registry-based cohort study lower eGDR, a surrogate marker for insulin resistance, was associated with a higher risk of first MI in individuals with diabetes. The impact of insulin resistance on MI risk was significantly greater in patients with type 1 diabetes compared to those with type 2 diabetes. Individuals with type 1 diabetes and pronounced insulin resistance (eGDR ≤4 and 4–6 mg/kg/min) had nearly double the risk of MI compared to individuals with type 2 diabetes and similar eGDR, even after adjustment for established cardiovascular risk factors.

Our findings are in line with previous evidence linking lower eGDR to macrovascular complications in both people with type 1 [13] and type 2 diabetes [11] and extend these observations by demonstrating a strong association between eGDR and first MI in a nationwide cohort. Similar associations have been reported with other indexes of insulin resistance and MI in people without diabetes, particularly in women [26]. Evidence in type 1 diabetes is more limited, although one study using the triglyceride-glucose (TyG)-index found that higher insulin resistance was associated with increased risk of MI, stroke, heart failure, and all-cause mortality [27]. Lower eGDR has also been linked to subclinical atherosclerosis in people with and without diabetes [28, 29]. Furthermore, insulin resistance, measured by hyperinsulinemic euglycemic clamp, predicts coronary artery calcification independent of glycemic control in people with type 1 diabetes [30].

In contrast, we did not observe a clear relationship between eGDR and post-MI mortality. Although mortality rates were significantly higher in individuals with the lowest compared to the highest eGDR, the CIs overlapped across intermediate eGDR categories. These findings suggest that insulin resistance is an important risk factor for first MI, although its influence on survival after MI may be limited. Results from a recent meta-analysis demonstrated that each unit increase in eGDR was associated with a 17% lower risk of CVD and a 16% reduction of all-cause mortality in people with type 1 diabetes [31]. In the current study, the lack of a similar graded association between eGDR and all-cause mortality post first MI may reflect limited statistical power, given the small number of post-MI deaths in people with type 1 diabetes. A comparable pattern was also observed in people with type 2 diabetes, despite a higher absolute number of events. It is possible that post-MI outcomes are more strongly influenced by acute management and other clinical factors than by baseline eGDR, or that insulin resistance primarily contributes to atherogenesis rather than post-infarction remodeling. Furthermore, 30- and 365-day mortality after first MI has declined over time for people with type 2 diabetes [8].

Although keeping glucose levels close to the normal range remains a cornerstone of diabetes management, other risk factors are important for predicting CVD. By integrating additional contributors of atherosclerosis, eGDR may help to distinguish a clinical risk phenotype, i.e. individuals with “double diabetes”. In a study of individuals with type 1 diabetes, eGDR but not metabolic syndrome (according to a harmonized definition from the main scientific societies), was associated with increased carotid plaque burden [29], supporting its potential added value for cardiovascular risk assessment. Nevertheless, whether eGDR accurately reflects insulin resistance remains debated. This index was originally validated in a small cohort of individuals with type 1 diabetes with sub-optimal glycemic control [10] and subsequent external validation studies have yielded inconsistent results [11, 32, 33]. Large-scale validation in type 2 diabetes is still lacking, however eGDR has shown good correlation with clamp-derived measures of insulin resistance in a small cohort of men with type 2 diabetes [11].

Visceral adiposity plays an important role in the development of insulin resistance [34], and waist circumference is generally considered a better marker of central fat distribution than BMI [35]. However, waist circumference is less frequently reported in the NDR and was therefore not available for alla individuals. BMI-based eGDR was used in the present study as it was more consistently recorded in the register, allowing inclusion of a larger study population and thereby increasing statistical precision. Nevertheless, in a previous study from our group examining eGDR based on waist circumference and its association with mortality in people with type 1 diabetes, sensitivity analysis showed similar results when using eGDR based on BMI [5].

The predictive value of eGDR beyond its individual components is debated, as they are themselves risk factors of CVD, making it difficult to assess their independent contributions. A recent study including 10,690 individuals with prediabetes and diabetes reported that eGDR had a better predictive power for CVD than its individual components and other insulin resistance indices, such as TyG and the triglyceride-to-high-density lipoprotein cholesterol ratio [36]. Similarly, in people without diabetes, eGDR has shown stronger predictive value for subclinical atherosclerosis than Homeostatic Model Assessment of Insulin Resistance (HOMA-IR) and TyG [28]. Insulin resistance may promote CVD through endothelial dysfunction, impaired nitric oxide bioavailability, oxidative stress, platelet activation, and vascular smooth muscle proliferation, as well as impaired myocardial energy metabolism and microvascular dysfunction, thereby increasing vulnerability to MI [37].

Comparing cardiovascular risk between individuals with type 1 diabetes and type 2 diabetes is challenging due to differences in pathophysiology. Age at onset and diabetes duration are key non-modifiable cardiovascular risk factors [38, 39]. After adjustment for these variables, HRs for first MI were attenuated in individuals with type 1 diabetes across all eGDR-categories (Supplemental Table S5), whereas a more modest attenuation was observed in individuals with type 2 diabetes. This suggests that cumulative exposure to diabetes (reflected by longer diabetes duration) partly explains the observed variation in cardiovascular risk between diabetes types. Diabetes duration and age of onset were not included in the final adjusted model to preserve this clinically meaningful distinction and because they act as structural time variables rather than conventional external confounders. Furthermore, it reduces potential bias arising from less reliable verification of diabetes onset in type 2 diabetes, where diagnosis is often delayed. As obesity and type 2 diabetes increasingly affect younger populations [40], a growing proportion of individuals with type 2 diabetes will reach older age with prolonged diabetes duration, resulting in duration distributions that more closely resemble those of type 1 diabetes. Consequently, the relative impact of diabetes duration as a distinguishing covariate may diminish over time. Prolonged diabetes duration increases cumulative glycemic exposure, an important contributor to cardiovascular risk [41].

### Strengths and limitations

A major strength of this study is its nationwide, population-based design, including a majority of people with diabetes in Sweden. Comprehensive linkage of high-quality national registers enabled adjustment for important cardiovascular risk factors, including age, sex, physical activity, lipid profile and kidney disease. Because HbA1c is a component of the eGDR formula, we did not adjust for HbA1c separately to avoid collinearity and overadjustment. However, the lack of adjustment for long-term glycemic variability is a limitation. Nevertheless, as in all observational studies, residual confounding cannot be excluded.

A limitation is the potential for misclassification bias in register-based data. eGDR was assumed to remain constant between measurements and was updated prospectively as new measurements became available, reducing regression dilution and avoiding allocation of person-time before complete eGDR could be defined. Consequently, individuals could contribute person-time to an incorrect eGDR category if metabolic status changed before updated measurements was recorded.

However, some components of eGDR, such as BMI and hypertension status, tend to change relatively slowly over time, whereas HbA1c may vary over shorter time periods. Measurements were typically updated during routine clinical follow up (median time 109 days), the overall risk of misclassification is likely limited and non-differential, since the timing of measurements is determined by routine care rather by the occurrence of MI. Moreover, the use of time-updated values better reflects current metabolic status than a baseline-only approach and may decrease the risk of regression dilution bias.

Misclassification of diabetes type cannot be excluded, particularly for LADA. In the NDR, LADA is typically recorded as type 1 diabetes, although some individuals with LADA may initially be registered as having type 2 diabetes of their adult onset and slower progression to insulin dependence. Individuals with LADA often have intermediate insulin resistance compared with classical type 1 diabetes group. This could overestimate the association between eGDR and MI in the type 1 diabetes cohort. However, the diagnostic validity of diabetes classification in the NDR has previously been shown to be high, suggesting that any potential misclassification is likely limited [42].

Outcome misclassification is likely limited, as MI diagnoses in the NPR have high validity [17]. Finally, more frequent health care contacts among individuals with poorly regulated diabetes may lead to more current information on covariates.

Broad population coverage in the NDR reduces selection bias, but regional differences may limit data completeness. As with any register data, not all variables are recorded, resulting in missing data. Generalizability may be restricted to health care systems comparable to Sweden, with universal, tax-funded health services with high-quality diabetes and CVD care provided at minimal individual cost. Another limitation is the lack of standardized cut-offs of eGDR, which could influence comparability across studies and clinical applicability.

## Conclusion

In summary, lower eGDR, a marker of insulin resistance, is strongly associated with first MI in people with diabetes, particularly in type 1 diabetes. Individuals with type 1 diabetes and marked insulin resistance, i.e. “double diabetes”, had nearly a two-fold risk of first MI compared to individuals with type 2 diabetes. These findings highlight the importance of early identification and targeted management, including intensified lifestyle interventions, adjunct pharmacological therapies, and aggressive primary prevention strategies [43]. Simple tools like eGDR may help identify high-risk individuals and guide precision CVD prevention in diabetes.

## Supplementary Information

Below is the link to the electronic supplementary material.

Supplementary Material 1

## Data Availability

Data are available from the national registers upon reasonable request and with appropriate approvals but are not publicly accessible due to privacy regulations.

## Declarations

### Author information

The abstract was presented as an Oral Presentation at the European Association for the Studies of Diabetes 61st Annual meeting in Vienna, Austria on the 18th of September 2025.

## Abbreviations

eGDR: estimated glucose disposal rate
eGFR: estimated glomerular filtration rate
ICD: International Classification of Diseases
LISA: Longitudinal Integration Database for Health Insurance and Labour Market Studies
NDR: National Diabetes Register
NPR: National Patient Register
PDR: Prescribed Drug Register
TyG index: Triglyceride-glucose index
