# Supplementary material for: Estimated glucose disposal rate and risk of first myocardial infarction in people with diabetes
Source: Cardiovasc Diabetol Endocrinol Rep. 2026 May 25;12:25. doi: 10.1186/s40842-026-00294-4 (PMC13200430; doi:10.1186/s40842-026-00294-4)
Supplement: Supplementary file 1 — Supplementary Material 1 [file 40842_2026_294_MOESM1_ESM.docx]

Supplemental Material

**Estimated glucose disposal rate and risk of first myocardial infarction in people with diabetes**

Linn Glynn, MD, Martina Persson MD, PhD, Tomas Andersson, B.Sc, Robin Hofmann MD, PhD, Thomas Nyström MD, PhD

**Figure S1.** Flowchart of the study population. BMI= body mass index. MI= myocardial infarction. NDR= National Diabetes Registry. NPR= National Patient Registry

Adults >18 years with diagnosis of diabetes in the NDR between 2006-2016

n= 802 964

Excluded: Lacking credible BMI values

n=104 073

Adults >18 years with diagnosis of diabetes in the NDR and credible BMI

n= 698 891

Excluded: Lacking complete registration of eGDR

n=3 828

Complete registration of eGDR_BMI_

n=695 063

Excluded: previous MI

n=50 937

Type 2 diabetes (NDR)

n= 570 230

Type 1 diabetes (NDR)

n= 46 155

Excluded: diagnosis ’’other diabetes’’

n=27 741

No previous MI

n=644 126

**Table S1.** Diagnosis according to International classification of diseases (ICD) 9^th^ Revision and 10^th^ Revision and Anatomical Therapeutic Chemical (ATC) codes for drugs

| **Diagnosis** | **ICD-code** |
| --- | --- |
| Acute myocardial infarction | I21 |
| Other forms of chronic ischemic heart disease | I25.8 |
| Chronic ischemic heart disease | 125.9 |
| Stroke | I60, I61, I62, I63, I64 |
| Chronic obstructive pulmonary disease | J44 |
| Heart failure | I50 |
| Atrial fibrillation | I48 |
| Peripheral arterial disease | I739 |
| Hypertension | I10 |
| Cancer | C |
| Coronary angiography | AF037 |
| Revascularization | FNG05, FNG02, FNA00, FNA10, FNC10, FNC20, FNC30, FNC40, FNG00 |
| Percutaneous coronary intervention | FNG05, FNG02, FNG00 |
| Coronary artery bypass graft | FNA00, FNA10, FNC10, FNC20, FNC30, FNC40 |
| **Drug** | **ATC-code** |
| Insulin all | A10A |
| Insulin long acting | A10AC. A10AD och A10AE |
| Insulin fast acting | A10AB |
| Metformin | A10BA |
| Sulfonylurea | A10BB |
| DPP-4 inhibitor | A10BH |
| GLP-1 receptor agonist | A10BJ |
| SGLT2-inhibitor | A10BK |

Dipeptidyl peptidase-4 (DPP4) inhibitors; glucagon-like peptide-1 (GLP-1) receptor agonists; sodium glucose cotransport-2 (SGLT2) inhibitors.

**Table S2.** Baseline characteristics of people with type 1 diabetes (T1D) at first complete registration of estimated glucose disposal rate (eGDR; mg/kg/min)

| **Baseline characteristics** |  |  |  |  |  |
| --- | --- | --- | --- | --- | --- |
| **eGDR** | ≤**4** | **4-6** | **6-8** | **≥8** | **all T1D** |
| n | 2 770 | 9 664 | 10 216 | 23 505 | 46 155 |
| Age, years, mean (±SD) | 47.6 (14.2) | 48.4 (15.1) | 38.3 (16.3) | 31.2 (12.3) | 37.3 (15.7) |
| Women, n (%) | 1303 (47.0) | 3781 (39.1) | 4766 (46.7) | 10 181 (43.3) | 20 031 (43.4) |
| Active smoker, n (%) | 402 (14.4) | 1491 (15.4) | 1770 (17.5) | 2916 (12.5) | 6579 (14.3) |
| BMI, kg/m^2^, mean (±SD) | 31.6 (4.9) | 26.4 (3.7) | 25.6 (4.2) | 23.4 (2.8) | 25.0 (4.0) |
| **Marital status, n (%)** |  |  |  |  |  |
| Married | 1147 (41.4) | 4356 (45.1) | 3110 (30.4) | 5280 (22.5) | 13 893 (30.1) |
| Separated/Single | 1533 (55.3) | 5025 (52.0) | 6950 (68.0) | 18 135 (77.2) | 31 643 (68.6) |
| Widowed | 91 (3.3) | 283 (2.9) | 154 (1.5) | 91 (0.4) | 619 (1.3) |
| **Education, n (%)** |  |  |  |  |  |
| Elementary school | 776 (28.2) | 2421 (25.2) | 3175 (31.5) | 7508 (32.4) | 13 880 (30.4) |
| Upper secondary school | 1462 (53.2) | 4891 (50.8) | 4593 (45.5) | 8997 (38.8) | 19 943 (43.7) |
| College level | 509 (18.5) | 2309 (24.0) | 2319 (23.0) | 6697 (28.9) | 11 834 (25.9) |
| **Blood lipids, mean (±SD)** |  |  |  |  |  |
| Total cholesterol, mmol/L | 5.06 (1.11) | 4.90 (0.99) | 4.81 (0.98) | 4.51 (0.89) | 4.70 (0.97) |
| HDL-cholesterol, mmol/L | 1.44 (0.45) | 1.61 (0.50) | 1.58 (0.50) | 1.58 (0.45) | 1.57 (0.47) |
| LDL-cholesterol, mmol/L | 2.91 (0.94) | 2.74 (0.83) | 2.72 (0.83) | 2.53 (0.76) | 2.65 (0.82) |
| Triglycerides, mmol/L | 1.69 (1.37) | 1.26 (0.97) | 1.19 (0.89) | 0.98 (0.65) | 1.14 (0.87) |
| Systolic blood pressure, mmHg, mean ± SD | 139 (17) | 137 (17) | 126 (15) | 119 (11) | 125 (16) |
| Diastolic blood pressure, mmHg, mean ± SD | 78 (10) | 76 (10) | 74 (9) | 71 (8) | 73 (9) |
| Creatinine, µmol/L, mean ± SD | 87.8 (53.9) | 87.9 (57.5) | 78.9 (55.3) | 72.7 (18.4) | 78.4 (42.4) |
| eGFR, ml/min, mean ± SD | 85.9 (31.5) | 85.8 (29.0) | 98.4 (30.7) | 102.6 (24.6) | 96.9 (28.5) |
| **Prior interventions, n (%)** |  |  |  |  |  |
| Coronary angiography | 15 (0.5) | 30 (0.3) | 13 (0.1) | 9 (0.0) | 67 (0.1) |
| Revascularization | 89 (3.2) | 212 (2.2) | 84 (0.8) | 20 (0.1) | 405 (0.9) |
| PCI | 38 (1.4) | 92 (1.0) | 33 (0.3) | 9 (<0.1) | 172 (0.4) |
| CABG | 57 (2.1) | 131 (1.4) | 55 (0.5) | 11 (<0.1) | 254 (0.6) |
| **History of comorbidities, n (%)** |  |  |  |  |  |
| Stroke | 103 (3.7) | 343 (3.5) | 155 (1.5) | 68 (0.3) | 669 (1.4) |
| Heart failure | 106 (3.8) | 178 (1.8) | 91 (0.9) | 15 (0.1) | 390 (0.8) |
| Atrial fibrillation | 64 (2.3) | 164 (1.7) | 72 (0.7) | 42 (0.2) | 342 (0.7) |
| Peripheral artery disease | 14 (0.5) | 76 (0.8) | 34 (0.3) | 2 (0.0) | 126 (0.3) |
| Hypertension | 1003 (36.2) | 2677 (27.7) | 1141 (11.2) | 373 (1.6) | 5194 (11.3) |
| COPD | 36 (1.3) | 67 (0.7) | 50 (0.5) | 25 (0.1) | 178 (0.4) |
| Cancer | 59 (2.1) | 168 (1.7) | 134 (1.3) | 144 (0.6) | 505 (1.1) |
| Cancer (within 2 years) | 17 (0.6) | 65 (0.7) | 57 (0.6) | 50 (0.2) | 189 (0.4) |
| **Treatments previous 180 days, n (%)** |  |  |  |  |  |
| Statin | 1272 (45.9) | 3572 (37.0) | 1695 (16.6) | 1312 (5.6) | 7851 (17.0) |
| ASA | 760 (27.4) | 2394 (24.8) | 1125 (11.0) | 542 (2.3) | 4821 (10.4) |
| P2Y12 | 38 (1.4) | 132 (1.4) | 59 (0.6) | 14 (0.1) | 243 (0.5) |
| Betablocker | 772 (27.9) | 2056 (21.3) | 813 (8.0) | 193 (0.8) | 3834 (8.3) |
| ACEi/ARB | 1679 (60.6) | 5248 (54.3) | 2102 (20.6) | 367 (1.6) | 9396 (20.4) |
| Diabetes onset age, years, mean (±SD) | 20.6 (12.2) | 19.4 (11.6) | 18.0 (10.9) | 18.1 (10.1) | 18.5 (10.8) |
| Diabetes duration, years, mean (±SD) | 25.9 (14.1) | 28.4 (14.9) | 19.8 (15.3) | 12.9 (11.6) | 18.4 (14.8) |
| HbA1c (mmol/mol), mean (±SD) | 80.0 (17.2) | 68.6 (15.9) | 70.0 (18.7) | 58.3 (12.2) | 64.3 (16.4) |
| HbA1c (DCCT %), mean (±SD) | 9.5 (3.7) | 8.4 (3.6) | 8.6 (3.9) | 7.5 (3.3) | 8.0 (3.7) |
| **Albuminuria, n (%)** |  |  |  |  |  |
| None | 1678 (58.5) | 6544 (66.6) | 8451 (84.4) | 21614 (95.4) | 38287 (84.4) |
| Microalbuminuria | 690 (24.1) | 2029 (20.6) | 1011 (10.1) | 843 (3.7) | 4573 (10.1) |
| Macroalbuminuria | 482 (16.8) | 1209 (12.3) | 513 (5.1) | 158 (0.7) | 2362 (5.2) |
| **Physical activity, times per week, n (%)** |  |  |  |  |  |
| Never | 602 (19.6) | 1175 (11.6) | 934 (9.4) | 1266 (5.9) | 3977 (8.9) |
| <1 times/week | 630 (20.6) | 1610 (15.9) | 1571 (15.9) | 2594 (12.1) | 6405 (14.4) |
| 1-2 times/week | 781 (25.5) | 2756 (27.2) | 2609 (26.4) | 5607 (26.2) | 11753 (26.4) |
| 3-5 times/week | 536 (17.5) | 2340 (23.1) | 2533 (25.6) | 6894 (32.2) | 12303 (27.7) |
| Daily | 515 (16.8) | 2247 (22.2) | 2242 (22.7) | 5025 (23.5) | 10029 (22.6) |
| **Diabetes treatment last 180 days, n (%)** |  |  |  |  |  |
| Diet only | 97 (3.5) | 450 (4.7) | 543 (5.3) | 1267 (5.4) | 2357 (5.1) |
| Insulin all | 2646 (95.5) | 9160 (94.8) | 9618 (94.1) | 22179 (94.4) | 43 603 (94.5) |
| Insulin long acting | 2275 (82.1) | 7736 (80.0) | 7854 (76.9) | 18382 (78.2) | 36 247 (78.5) |
| Insulin fast acting | 2341 (84.5) | 8251 (85.4) | 8862 (86.7) | 20924 (89.0) | 40 378 (87.5) |
| Metformin | 337 (12.2) | 390 (4.0) | 351 (3.4) | 333 (1.4) | 1411 (3.1) |
| Sulfonylurea | 38 (1.4) | 64 (0.7) | 29 (0.3) | 33 (0.1) | 164 (0.4) |
| DPP-4 inhibitor | 10 (0.4) | 6 (0.1) | 9 (0.1) | 27 (0.1) | 52 (0.1) |
| GLP-1 receptor agonist | 13 (0.5) | 16 (0.2) | 8 (0.1) | 12 (0.1) | 49 (0.1) |
| SGLT2-inhibitor | 1 (<0.1) | 4 (<0.1) | 3 (<0.1) | 5 (<0.1) | 13 (<0.1) |

ASA, Acetylsalicylic acid; ACE-I, Angiotensin-converting-enzyme inhibitor; ARB, Angiotensin II receptor-blocker; BMI, Body mass index; CABG, Coronary artery bypass graft; COPD, chronic obstructive lung disease; DPP-4 inhibitor, Dipeptidylpeptidas inhibitor; eGFR, Estimated glomerular filtration rate; GLP-1, Glucagon like peptide-1 receptor agonist; HbA1c, Glycated haemoglobin 1c; HDL-Cholesterol, High-density lipoprotein-Cholesterol; LDL-Cholesterol, Low-density lipoprotein-Cholesterol, PCI, percutaneous coronary intervention; P2Y12, P2Y12-inhibitors; SGLT2-inhibitor, Sodium-glucose transport 2-inhibitor

**Table S3.** Baseline characteristics of people with type 2 diabetes (T2D) at first complete registration of estimated glucose disposal rate (mg/kg/min)

| **Baseline characteristics** |  |  |  |  |  |
| --- | --- | --- | --- | --- | --- |
| **eGDR** | **≤4** | **4-6** | **6-8** | **≥8** | **all T2D** |
| n | 118 885 | 235 797 | 131 167 | 84 381 | 570 230 |
| Age, years, mean (±SD) | 61.6 (12.0) | 66.7 (11.7) | 65.9 (13.7) | 60.9 (13.2) | 64.6 (12.7) |
| Women, n (%) | 55 686 (46.8) | 99 789 (42.3) | 58 326 (44.5) | 33 562 (39.8) | 247 363 (43.4) |
| Active smoker, n (%) | 17 466 (15.1) | 32 260 (13.8) | 20 872 (16.3) | 14 793 (18.4) | 85 391 (15.3) |
| BMI, kg/m^2^, mean (±SD) | 36.7 (5.7) | 30.2 (3.7) | 27.4 (4.8) | 26.7 (3.4) | 30.4 (5.7) |
| **Marital status, n (%)** |  |  |  |  |  |
| Married | 56 819 (47.8) | 128 075 (54.3) | 70 308 (53.6) | 47 145 (55.9) | 302 347 (53.0) |
| Separated/Single | 50 676 (42.6) | 76 926 (32.6) | 43 608 (33.3) | 30 796 (36.5) | 202 006 (35.4) |
| Widowed | 11 364 (9.6) | 30 822 (13.1) | 17 227 (13.1) | 6456 (7.6) | 65 869 (11.6) |
| **Education, n (%)** |  |  |  |  |  |
| Elementary school | 42 233 (36.3) | 91 230 (39.4) | 48 421 (37.7) | 25 959 (31.4) | 207 843 (37.2) |
| Upper secondary school | 54 476 (46.8) | 98 862 (42.7) | 54 239 (42.2) | 36 181 (43.7) | 243 758 (43.6) |
| College level | 19 656 (16.9) | 41 291 (17.8) | 25 913 (20.2) | 20 612 (24.9) | 107 472 (19.2) |
| **Blood lipids, mean (±SD)** |  |  |  |  |  |
| Total cholesterol, mmol/L | 5.10 (1.20) | 4.98 (1.13) | 5.00 (1.14) | 5.08 (1.11) | 5.03 (1.15) |
| HDL-cholesterol, mmol/L | 1.16 (0.34) | 1.25 (0.37) | 1.33 (0.43) | 1.31 (0.42) | 1.26 (0.39) |
| LDL-cholesterol, mmol/L | 2.97 (1.03) | 2.91 (1.00) | 2.95 (1.00) | 3.08 (0.98) | 2.95 (1.01) |
| Triglycerides, mmol/L | 2.41 (1.90) | 1.97 (1.33) | 1.76 (1.33) | 1.69 (1.21) | 1.98 (1.48) |
| Systolic blood pressure, mmHg, mean ± SD | 142 (17) | 140 (17) | 136 (17) | 126 (11) | 138 (17) |
| Diastolic blood pressure, mmHg, mean ± SD | 83 (11) | 80 (10) | 77 (9) | 75 (8) | 79 (10) |
| Creatinine, µmol/L, mean ± SD | 76.4 (28.6) | 80.6 (29.4) | 78.6 (29.1) | 74.5 (22.7) | 78.4 (28.4) |
| eGFR, ml/min, mean ± SD | 86.8 (26.6) | 80.7 (23.5) | 83.1 (24.7) | 88.7 (22.6) | 83.6 (24.5) |
| **Prior interventions, n (%)** |  |  |  |  |  |
| Coronary angiography | 2573 (2.2) | 5448 (2.3) | 2493 (1.9) | 839 (1.0) | 11 353 (2.0) |
| Revascularization | 3109 (2.6) | 8221 (3.5) | 3377 (2.6) | 889 (1.1) | 15 596 (2.7) |
| PCI | 1716 (1.4) | 4331 (1.8) | 1775 (1.4) | 483 (0.6) | 8305 (1.5) |
| CABG | 1531 (1.3) | 4268 (1.8) | 1752 (1.3) | 453 (0.5) | 8004 (1.4) |
| **History of comorbidities, n (%)** |  |  |  |  |  |
| Stroke | 5642 (4.7) | 14 778 (6.3) | 7989 (6.1) | 2719 (3.2) | 31 128 (5.5) |
| Heart failure | 7004 (5.9) | 11 315 (4.8) | 5187 (4.0) | 1428 (1.7) | 24 934 (4.4) |
| Atrial fibrillation | 8781 (7.4) | 18 948 (8.0) | 9476 (7.2) | 2644 (3.1) | 39 849 (7.0) |
| Peripheral artery disease | 445 (0.4) | 1385 (0.6) | 831 (0.6) | 203 (0.2) | 2864 (0.5) |
| Hypertension | 44 410 (37.4) | 83 351 (35.3) | 34 810 (26.5) | 7117 (8.4) | 169 688 (29.8) |
| COPD | 4118 (3.5) | 6913 (2.9) | 3842 (2.9) | 1977 (2.3) | 16 850 (3.0) |
| Cancer | 6080 (5.1) | 15242 (6.5) | 8840 (6.7) | 4974 (5.9) | 35 136 (6.2) |
| Cancer (within 2 years) | 1630 (1.4) | 4374 (1.9) | 2712 (2.1) | 1694 (2.0) | 10 410 (1.8) |
| **Treatments previous 180 days, n (%)** |  |  |  |  |  |
| Statin | 48 885 (41.1) | 106 874 (45.3) | 49 760 (37.9) | 24 068 (28.5) | 229 587 (40.3) |
| ASA | 29 084 (24.5) | 69 957 (29.7) | 32 344 (24.7) | 11 433 (13.5) | 142 818 (25.0) |
| P2Y12 | 2004 (1.7) | 5223 (2.2) | 2707 (2.1) | 958 (1.1) | 10 892 (1.9) |
| Betablocker | 48 301 (40.6) | 98 044 (41.6) | 40 561 (30.9) | 7391 (8.8) | 194 297 (34.1) |
| ACEi/ARB | 74 007 (62.3) | 141 590 (60.0) | 54 545 (41.6) | 8087 (9.6) | 278 229 (48.8) |
| Diabetes onset age, years, mean (±SD) | 56.9 (12.1) | 61.8 (12.0) | 61.6 (13.5) | 57.2 (13.2) | 60.1 (12.8) |
| Diabetes duration, years, mean (±SD) | 4.6 (6.8) | 4.8 (6.7) | 4.2 (6.2) | 3.5 (5.4) | 4.4 (6.4) |
| HbA1c (mmol/mol), mean (±SD) | 67.0 (21.2) | 52.8 (13.1) | 49.8 (13.5) | 47.5 (8.8) | 54.3 (16.3) |
| HbA1c (DCCT %), mean (±SD) | 8.3 (4.1) | 7.0 (3.4) | 6.7 (3.4) | 6.5 (3.0) | 7.1 (3.6) |
| **Albuminuria, n (%)** |  |  |  |  |  |
| None | 76931 (72.6) | 175134 (78.3) | 98940 (82.1) | 63965 (89.0) | 414970 (79.5) |
| Microalbuminuria | 20031 (18.9) | 34037 (15.2) | 15403 (12.8) | 5928 (8.2) | 75399 (14.4) |
| Macroalbuminuria | 8518 (8.0) | 13430 (6.0) | 5575 (4.6) | 1677 (2.3) | 29200 (5.6) |
| **Physical activity, times per week, n (%)** |  |  |  |  |  |
| Never | 26397 (23.5) | 33451 (14.8) | 16193 (13.4) | 7512 (10.1) | 83553 (15.7) |
| <1 times/week | 19911 (17.7) | 28739 (12.7) | 13122 (10.8) | 7018 (9.5) | 68790 (12.9) |
| 1-2 times/week | 24226 (21.6) | 48012 (21.3) | 23098 (19.1) | 13942 (18.8) | 109278 (20.5) |
| 3-5 times/week | 19620 (17.5) | 49628 (22.0) | 27028 (22.3) | 18706 (25.3) | 114982 (21.6) |
| Daily | 22040 (19.6) | 65600 (29.1) | 41630 (34.4) | 26848 (36.3) | 156118 (29.3) |
| **Diabetes treatment last 180 days, n (%)** |  |  |  |  |  |
| Diet only | 28961 (24.4) | 84187 (35.7) | 56252 (42.9) | 36696 (43.5) | 206096 (36.1) |
| Insulin all | 28613 (24.1) | 38141 (16.2) | 16294 (12.4) | 9849 (11.7) | 92897 (16.3) |
| Insulin long acting | 20270 (17.1) | 24901 (10.6) | 10502 (8.0) | 6699 (7.9) | 62372 (10.9) |
| Insulin fast acting | 8929 (7.5) | 10641 (4.5) | 4870 (3.7) | 3385 (4.0) | 27825 (4.9) |
| Metformin | 74311 (62.5) | 119014 (50.5) | 56929 (43.4) | 37451 (44.4) | 287705 (50.5) |
| Sulfonylurea | 11990 (10.1) | 28204 (12.0) | 13642 (10.4) | 7318 (8.7) | 61154 (10.7) |
| DPP-4 inhibitor | 2762 (2.3) | 4094 (1.7) | 2017 (1.5) | 1395 (1.7) | 10268 (1.8) |
| GLP-1 receptor agonist | 1535 (1.3) | 1152 (0.5) | 500 (0.4) | 278 (0.3) | 3465 (0.6) |
| SGLT2-inhibitor | 812 (0.7) | 971 (0.4) | 472 (0.4) | 330 (0.4) | 2585 (0.5) |

ASA, Acetylsalicylic acid; ACE-I, Angiotensin-converting-enzyme inhibitor; ARB, Angiotensin II receptor-blocker; BMI, Body mass index; CABG, Coronary artery bypass graft; COPD, chronic obstructive lung disease; DPP-4 inhibitor, Dipeptidylpeptidas inhibitor; eGFR, Estimated glomerular filtration rate; GLP-1, Glucagon like peptide-1 receptor agonist; HbA1c, Glycated haemoglobin 1c; HDL-Cholesterol, High-density lipoprotein-Cholesterol; LDL-Cholesterol, Low-density lipoprotein-Cholesterol, PCI, percutaneous coronary intervention; P2Y12, P2Y12-inhibitors; SGLT2-inhibitor, Sodium-glucose transport 2-inhibitor

**Table S4.** Crude and age standardized incidence rates for first myocardial infarction (MI) stratified by sex and diabetes type (T1D= type 1 diabetes; T2D=type 2 diabetes

|  | | **Men** | | | | **Women** | | | |
| --- | --- | --- | --- | --- | --- | --- | --- | --- | --- |
|  | **eGDR** | **n MI** | **p-yrs** | **Crude rate per 10 000** | **Age standardized rate per 10 000** | **n MI** | **p-yrs** | **Crude rate per 10 000** | **Age standardized rate per 10 000** |
| **T1D** | ≤4 | 236 | 30 922 | 76.3 (67.2-86.7) | 140.6 (112.1-176.3) | 205 | 26 065 | 78.6 (68.6-90.2) | 137.6 (109.1-173.5) |
|  | 4-6 | 615 | 104 724 | 58.7 (54.3-63.6) | 112.0 (100.2-125.3) | 434 | 64 730 | 67.0 (61.0-73.7) | 153.3 (132.2-177.9) |
|  | 6-8 | 208 | 66 343 | 31.4 (27.4-35.9) | 91.6 (76.4-109.7) | 188 | 59 218 | 31.7 (27.5-36.6) | 92.6 (74.8-114.7) |
|  | ≥8 | 60 | 110 436 | 5.4 (4.2-7.0) | 46.5 (21.7-99.6) | 51 | 94 051 | 5.4 (4.1-7.1) | 59.0 (25.3-137.3) |
| **T2D** | ≤4 | 4 638 | 490 646 | 94.5 (91.8-97.3) | 101.6 (98.6-104.6) | 3 375 | 460 006 | 73.4 (70.9-75.9) | 76.9 (74.3-79.6) |
|  | 4-6 | 11 316 | 1 207 620 | 93.7 (92.0-95.4) | 84.4 (82.8-86.1) | 6 004 | 873 320 | 68.7 (67.0-70.5) | 59.3 (57.8-60.9) |
|  | 6-8 | 4 654 | 550 640 | 84.5 (82.1-87.0) | 71.3 (69.2-73.5) | 2 796 | 456 038 | 61.3 (59.1-63.6) | 50.3 (48.4-52.3) |
|  | ≥8 | 1 045 | 237 423 | 44.0 (41.4-46.8) | 52.3 (49.0-55.7) | 409 | 169 224 | 24.2 (21.9-26.6) | 34.6 (31.2-38.3) |

**Table S5.** Hazard ratios with 95 % CIs for first myocardial infarction, adjusted for each covariate separately.

|  | Type 1 diabetes | | | | Type 2 diabetes | | | |
| --- | --- | --- | --- | --- | --- | --- | --- | --- |
| eGDR (mg/kg/min) | **≤4** | **4-6** | **6-8** | **≥8** | **≤4** | **4-6** | **6-8** | **≥8** |
| HR Physical activity | 3.91 (3.51-4.35) | 2.76 (2.54-2.99) | 1.95 (1.74-2.18) | 0.71 (0.58-0.87) | 1.99 (1.89-2.11) | 1.66 (1.57-1.75) | 1.43 (1.35-1.51) | 1 |
| HR HDL | 4.53 (4.07-5.04) | 3.31 (3.05-3.59) | 2.43 (2.17-2.72) | 0.88 (0.72-1.07) | 2.02 (1.91-2.14) | 1.63 (1.55-1.72) | 1.44 (1.36-1.53) | 1 |
| HR Albuminuria | 3.68 (3.30-4.10) | 2.60 (2.40-2.82) | 1.86 (1.66-2.08) | 0.73 (0.60-0.89) | 1.99 (1.88-2.10) | 1.62 (1.53-1.71) | 1.39 (1.31-1.47) | 1 |
| HR TG | 4.36 (3.91-4.85) | 3.02 (2.79-3.27) | 2.12 (1.89-2.37) | 0.78 (0.64-0.96) | 2.06 (1.95-2.18) | 1.66 (1.57-1.75) | 1.43 (1.35-1.51) | 1 |
| HR eGFR | 3.82 (3.43-4.25) | 2.62 (2.42-2.84) | 1.83 (1.63-2.04) | 0.67 (0.55-0.81) | 2.09 (1.97-2.21) | 1.65 (1.56-1.74) | 1.41 (1.33-1.49) | 1 |
| HR LDL | 4.68 (4.20-5.21) | 3.17 (2.93-3.44) | 2.20 (1.97-2.46) | 0.79 (0.65-0.96) | 2.34 (2.21-2.47) | 1.79 (1.70-1.89) | 1.49 (1.41-1.58) | 1 |
| HR age of onset | 2.30 (2.05-2.57) | 1.44 (1.32-1.58) | 0.97 (0.87-1.10) | 0.41 (0.33-0.50) | 2.05 (1.94-2.17) | 1.65 (1.57-1.74) | 1.42 (1.34-1.50) | 1 |
| HR duration | 2.22 (1.98-2.48) | 1.39 (1.27-1.51) | 0.93 (0.83-1.05) | 0.40 (0.33-0.49) | 2.04 (1.93-2.16) | 1.65 (1.56-1.74) | 1.42 (1.34-1.51) | 1 |

HDL= high-density lipoprotein-Cholesterol; LDL= low-density lipoprotein-Cholesterol; TG= triglycerides; eGFR= estimated glomerular filtration rate

**
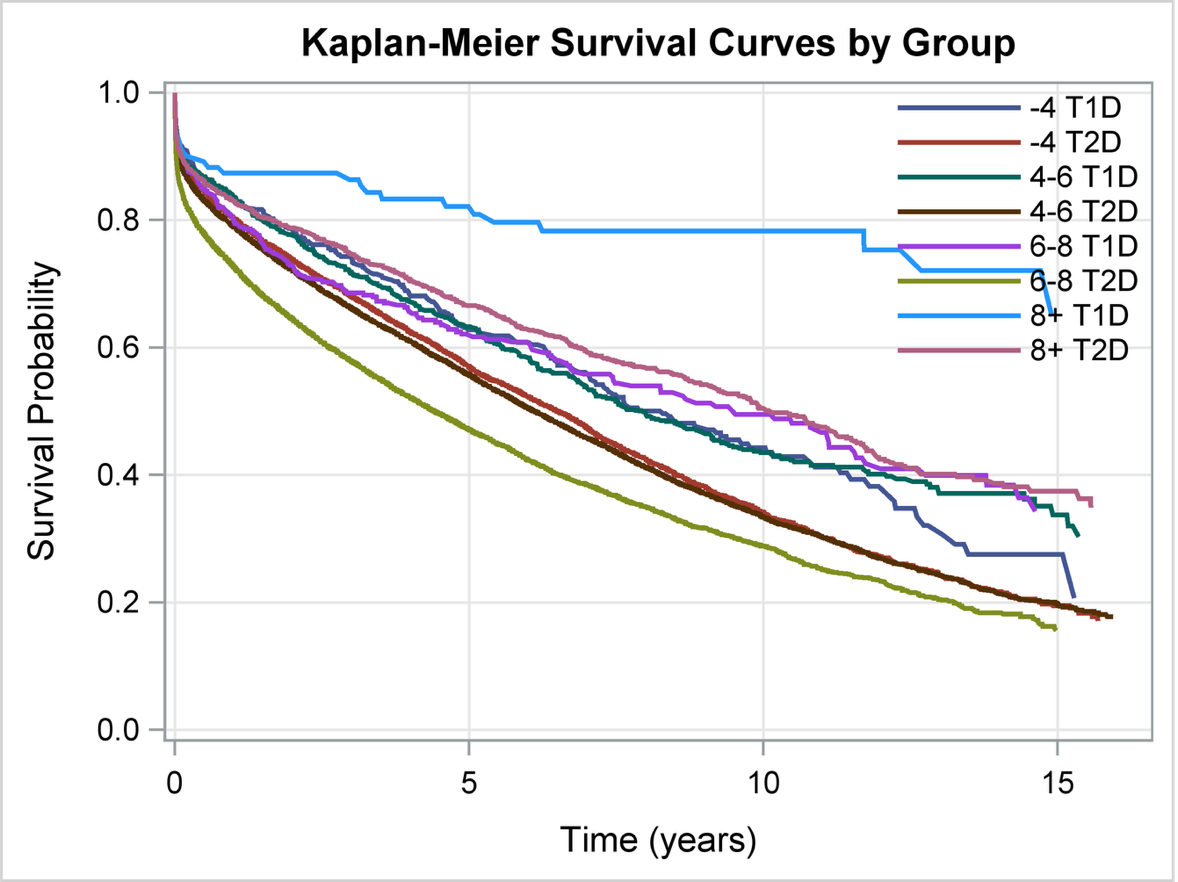
**

**Figure S2.** Estimated Kaplan-Meier survival curve of mortality after first myocardial infarction per estimated glucose disposal rate and diabetes type (T1D=type 1 diabetes; T2D=type 2 diabetes).

**Table S6.** Sex stratified mortality rates after first-time myocardial infarction by diabetes type and estimated glucose disposal rate (eGDR) in mg/kg/min

|  | | **Men** | | | **Women** | | |
| --- | --- | --- | --- | --- | --- | --- | --- |
|  | **eGDR** | **n dead** | **p-yrs** | **Rate per 100 p-yrs** | **n dead** | **p-yrs** | **Rate per 100 p-yrs** |
| **T1D** | ≤4 | 119 | 1 183 | 10.1 (8.4-12.0) | 90 | 1 098 | 8.2 (6.7-10.1) |
|  | 4-6 | 279 | 3 429 | 8.1 (7.2-9.2) | 224 | 2 194 | 10.2 (9.0-11.6) |
|  | 6-8 | 93 | 1 056 | 8.8 (7.2-10.8) | 88 | 1 008 | 8.7 (7.1-10.8) |
|  | >8 | 12 | 449 | 2.7 (1.5-4.7) | 13 | 368 | 3.5 (2.1-6.1) |
| **T2D** | ≤4 | 2 289 | 21 297 | 10.7 (10.3-11.2) | 1 930 | 14 482 | 13.3 (12.7-13.9) |
|  | 4-6 | 5 594 | 51 503 | 10.9 (10.6-11.1) | 3 708 | 23 938 | 15.5 (15.0-16.0) |
|  | 6-8 | 2 580 | 18 508 | 13.9 (13.4-14.5) | 1 855 | 9 772 | 19.0 (18.1-19.9) |
|  | >8 | 409 | 6 205 | 6.6 (6.0-7.3) | 219 | 1 857 | 11.8 (10.3-13.5) |
